# Commentary: Transforming growth factor serum concentrations in patients with proven non-syndromic aortopathy

**DOI:** 10.3389/fcvm.2023.1097201

**Published:** 2023-02-02

**Authors:** Nimrat Grewal, Robert Klautz, Robert E. Poelmann

**Affiliations:** ^1^Department of Cardiothoracic Surgery, Amsterdam University Medical Center, Amsterdam, Netherlands; ^2^Department of Cardiothoracic Surgery, Leiden University Medical Center, Leiden, Netherlands; ^3^Department of Anatomy and Embryology, Leiden University Medical Center, Leiden, Netherlands; ^4^Institute of Biology, Animal Sciences and Health, Leiden University, Leiden, Netherlands; ^5^Department of Cardiology, Leiden University Medical Center, Leiden, Netherlands

**Keywords:** bicuspid and tricuspid aortic valve, transforming growth factor β1 (TGF-β1), aortopathy, biomarkers, aneurysm thoracic surgery

## Introduction

This commentary builds on the recently published original paper of Karalko et al. (1). The authors presented an interesting study aiming at identifying a potential diagnostic marker in non-syndromic patients with aortic dilatation. The aim of this commentary is to advance the debate currently going on identifying high risk bicuspid aortic valve patients and whether serum transforming growth factor beta (TGF-β) is a suitable marker.

### How can we identify bicuspid aortic valve patients at risk for future aortopathy?

Patients with a bicuspid aortic valve (BAV) have a 70-fold increased risk to develop a thoracic aortic aneurysm or dissection as compared to patients with a normal tricuspid aortic valve (TAV) (2). Thoracic aortic aneurysms and dissections are life-threatening events but preventable if individuals-at-risk are identified and properly managed. Hereditary forms of thoracic aortic aneurysms can be subdivided into non- and syndromic. Syndromic forms of thoracic aortic aneurysms occur in patients affected by connective tissue disorders such as Marfan, vascular Ehlers-Danlos and Loeys-Dietz syndrome, in all of which a defective TGF-β signaling has been described. BAV disease is the most common non-syndromic cardiac anomaly with a sharply increased risk for thoracic aortopathy. Even though many histopathological similarities have been described between the BAV and syndromic forms of thoracic aortic aneurysms and dissections the exact role of TGF-β is not entirely understood in the BAV (3, 4).

As thoracic aortic aneurysms do not have preceding symptoms, the first manifestation often is chest pain due to a dissected aorta. Current risk stratification and treatment strategy of patients with an increased risk for aortopathy is till date solely based on the aortic diameter. Nonetheless, most of the life-threatening aortic complications cannot be prevented by following the guidelines-based geometrical cut off for surgical treatment of the aorta. Therefore, the until date used maximum aortic diameter alone cannot be implemented as a reliable parameter for risk stratification and subsequent treatment of aortopathy. Risk stratification based on the structural abnormalities of the ascending aortic wall should therefore be considered to identify patients at risk for future aortic complications. In the past decade over 20 papers have been published about the development of personalized prognostic markers in bicuspid aortopathy. Despite vigorous research efforts, no biomarker or imaging tool has, however, till date been established to identify BAV patients at risk for thoracic aortic complications. The major drawback in most of the studies is an extensive investigation of aneurysmal tissue, instead of focusing on rarities within the non-dilated aortic specimen which could predict future aortopathy. Furthermore, the raphe position, aortic valve pathology, the role of hemodynamics and longitudinal follow-up are factors which have not gained enough attention while searching for prognostic markers (5, 6). To study prognostic markers for future aortic complications similarities between (a subset) of non-dilated and dilated ascending aortic wall samples should be identified (7). Markers should be correlated to clinical characteristics and most importantly followed longitudinally. Biomarkers can be tissue specific, or derivates in blood. Karalko et al. (1) describe in their study the expression of a potential diagnostic marker in blood.

### Transforming growth factor beta as a diagnostic/prognostic marker

In the paper of Karalko et al. (1), TGF-β1 levels in plasma were measured in 50 patients who had undergone cardiac surgery. Bicuspid (BAV) and tricuspid (TAV) aortic valve patients, with and without an aortic dilatation were enrolled in the study, a reference group of 40 volunteers was also included. In their study Karalko et al. (1) conclude that the serum TGF-β1 concentration is significantly changed in non- and syndromic thoracic aortic aneurysms, as well as patients with a BAV. This is an interesting paper aiming at identifying potential biomarkers to predict future aortopathy in BAV patients, but there are some limitations which should be considered while interpreting the presented data.

Bicuspid aortopathy and thoracic aortic aneurysm formation are very heterogenous disorders with regard to the etiology and natural history. As the authors also suggest themselves, the study has a very small sample size which makes it difficult to draw conclusions about a specific clinical subset of patients. Demographic and cardiovascular risk factors age, sex, body mass index, hypertension and dyslipidaemia were significantly distinct between the non- and dilated ascending aortic group, however, findings were not corrected for these variables. Moreover, it has previously been shown that in patients with aortic regurgitation the proximal aorta shows increased inflammation and medial degeneration as compared to patients with aortic stenosis (8). In the presented study, significantly more regurgitant BAV patients were included in the dilated ascending aortic wall group, which could partly explain the encountered altered expression of TGF-β1 levels as TGF-β stands central among inflammatory and extracellular matrix regulatory cytokines and possesses both important immunomodulatory and remodeling activities (9). The role of shear stress on the aortic wall should also be considered, as it has been shown that in BAV patients with aortic valve stenosis, the amount of TGF-β in the aortic wall correlates well with the levels of shear stress on the wall (10). Therefore, to reach more meaningful conclusions, a large sample size with patients matched to the demographics, cardiovascular risk factors and aortic valve pathology is crucial as well as the correlation between plasma and tissue levels of TGF-β expression.

Transforming growth factor beta is an important signaling pathway involved in many cellular pathways that play key roles for tissue maintenance. In recent years numerous studies have addressed TGF-β1 levels in plasma to monitor disease progression and effects of treatment. Diseases which were studied ranged from dermatological conditions such as psoriasis (11), to tumors such as multiple myeloma and breast cancer (12, 13). In all these papers the serum TGF-β1 expression was advocated as a marker of disease activity. An increased TGF-β1 expression has even been described in healthy subjects, as a marker of increased cortical thickness (14). Considering the broad role of TGF-β1 in the tissue homeostasis and alterations in expression levels in multiple disease states, this signaling pathway needs to be critically studied for its specificity for the ascending aortic wall as the study of Karalko et al. (1) suggests. We have clinical examples of inflammatory markers which also lack specificity for a particular inflammatory disease process but are still highly useful to monitor the progression and therapeutic response in patients with a given and active disease such as c-reactive protein and erythrocyte sedimentation rate. To draw conclusions about the role of a widely expressed protein such as TGF-β1 in monitoring a specified disease process several factors need be addressed which the authors did not mention in their study, such as the extent of overexpression and/or systemic release in thoracic aortopathy, the rarity and/or ability to exclude other potentially confounding disease processes and the potential use of complementary markers to enhance specificity.

Furthermore, the active and latent forms of TGF-β1 and platelet-derived TGF-β1 are not distinguished in the results (1). Studies which the authors refer to in their discussion also focused on serum levels of TGF-β1 as a potential marker for future aortopathy, however, they had correlated their findings to the local expression of TGF-β1 in the ascending aortic wall (15, 16). Moreover, one of these studies for the first time showed a relation between the levels of the identified biomarker and a clinical outcome (i.e., rate of aneurysm diameter progression in longitudinal observation) (16). Several features which are highly characteristic for BAV patients, such as dedifferentiated vascular smooth muscle cells, a significantly thinner intimal layer and a lack of atherosclerosis are indeed related to a defective TGF-β signaling (17–20). TGF-β signaling, for instance, plays pivotal roles in smooth muscle cell differentiation during vascular development as well as phenotypic switching in disease states (21). TGF-β further plays a central role in (physiological) thickening of the intimal layer (20), due to a process of low-grade injury and repair (22, 23). The characteristic BAV findings, which can be related to a defective TGF-β signaling resulting in a decreased plasma level, are, however, generic to all BAV patients and cannot be applied to identify the high-risk subset.

In summary, when searching for potential prognostic markers in the highly heterogenous BAV aortopathy patients we recommend a robust matched study population, serial measurements of the biomarkers and most importantly a correlation with the clinical phenotype.

## Conclusion

Patients with a BAV have an increased risk to develop thoracic aortic complications. Karalko et al. (1) are commendable for their effort to study TGF-β as a diagnostic marker to identify high risk BAV patients. In this paper we argue that the presented study has considerable limitations which complicates the understanding of the conclusions regarding plasma TGF-β expression. To develop a patient tailored risk stratification we recommend studying the ascending aortic wall properties of non-dilated BAV patients. After identification of tissue specific markers which are pathologic in a subset of patients susceptible for future complications, derivates should be studied in plasma. Longitudinal follow up of the identified markers is the key in establishing patient tailored risk stratification.

## Author contributions

NG wrote the first draft of the manuscript. RK and RP contributed to manuscript revision, read, and approved the submitted version. All authors contributed to the article and approved the submitted version.

## References

[1] Karalko M Pojar M Zaloudkova L Stejskal V Timbilla S Brizova P Transforming growth factor serum concentrations in patients with proven non-syndromic aortopathy. *Front Cardiovasc Med.* (2022) 9:980103. 10.3389/fcvm.2022.980103 36148051 PMC9485481

[2] Grewal N Velders BJJ Gittenberger-de Groot AC Poelmann R Klautz RJM Van Brakel TJ A systematic histopathologic evaluation of Type-A aortic dissections implies a uniform multiple-hit causation. *J Cardiovasc Dev Dis.* (2021) 8:12. 10.3390/jcdd8020012 33513898 PMC7911401

[3] Grewal N Marie-José G DeRuiter MC Klautz RJM Poelmann RE Mulder BJM Aortopathy in bicuspid aortic valve and marfan syndrome is characterized by a lack of activation potential of the epicardium in the ascending aorta. *Int J Pathol Clin Res.* (2017) 3:051. 10.23937/2469-5807/1510051

[4] Grewal N Gittenberger-de Groot AC. Pathogenesis of aortic wall complications in Marfan syndrome. *Cardiovasc Pathol.* (2018) 33:62–9. 10.1016/j.carpath.2018.01.005 29433109

[5] Grewal N Girdauskas E Deruiter M Goumans MJ Lindeman JH Disha K The effects of hemodynamics on the inner layers of the aortic wall in patients with a bicuspid aortic valve. *Integrat Mol Med.* (2017) 4:1–7. 10.15761/IMM.1000308

[6] Koenraadt WM Grewal N Gaidoukevitch OY DeRuiter MC Gittenberger-de Groot AC Bartelings MM The extent of the raphe in bicuspid aortic valves is associated with aortic regurgitation and aortic root dilatation. *Neth Heart J.* (2016) 24:127–33. 10.1007/s12471-015-0784-4 26758507 PMC4722007

[7] Grewal N Gittenberger-de Groot AC DeRuiter MC Klautz RJ Poelmann RE Duim S Bicuspid aortic valve: phosphorylation of c-Kit and downstream targets are prognostic for future aortopathy. *Eur J Cardiothorac Surg.* (2014) 46:831–9. 10.1093/ejcts/ezu319 25161185

[8] Sequeira Gross TM Lindner D Ojeda FM Neumann J Grewal N Kuntze T Comparison of microstructural alterations in the proximal aorta between aortic stenosis and regurgitation. *J Thorac Cardiovasc Surg.* (2021) 162:1684–95. 10.1016/j.jtcvs.2020.03.002 32386768

[9] Yang YC Zhang N Van Crombruggen K Hu GH Hong SL Bachert C. Transforming growth factor-beta1 in inflammatory airway disease: a key for understanding inflammation and remodeling. *Allergy.* (2012) 67:1193–202. 10.1111/j.1398-9995.2012.02880.x 22913656

[10] Guzzardi DG Barker AJ van Ooij P Malaisrie SC Puthumana JJ Belke DD Valve-related hemodynamics mediate human bicuspid aortopathy: insights from wall shear stress mapping. *J Am Coll Cardiol.* (2015) 66:892–900. 10.1016/j.jacc.2015.06.1310 26293758 PMC4545965

[11] Nockowski P Szepietowski JC Ziarkiewicz M Baran E. Serum concentrations of transforming growth factor beta 1 in patients with psoriasis vulgaris. *Acta Dermatovenerol Croat.* (2004) 12:2–6. 15072741

[12] Ciftci R Tas F Yasasever CT Aksit E Karabulut S Sen F High serum transforming growth factor beta 1 (TGFB1) level predicts better survival in breast cancer. *Tumour Biol.* (2014) 35:6941–8. 10.1007/s13277-014-1932-y 24740564

[13] Kyrtsonis MC Repa C Dedoussis GV Mouzaki A Simeonidis A Stamatelou M Serum transforming growth factor-beta 1 is related to the degree of immunoparesis in patients with multiple myeloma. *Med Oncol.* (1998) 15:124–8. 10.1007/bf02989591 9789221

[14] Piras F Salani F Bossù P Caltagirone C Spalletta G. High serum levels of transforming growth factor β1 are associated with increased cortical thickness in cingulate and right frontal areas in healthy subjects. *J Neuroinflammation.* (2012) 9:42. 10.1186/1742-2094-9-42 22373370 PMC3359165

[15] Gomez D Al Haj Zen A Borges LF Philippe M Gutierrez PS Jondeau G Syndromic and non-syndromic aneurysms of the human ascending aorta share activation of the Smad2 pathway. *J Pathol.* (2009) 218:131–42. 10.1002/path.2516 19224541

[16] Forte A Bancone C Cobellis G Buonocore M Santarpino G Fischlein TJM A possible early biomarker for bicuspid aortopathy: circulating transforming growth factor β-1 to soluble endoglin ratio. *Circ Res.* (2017) 120:1800–11. 10.1161/circresaha.117.310833 28420669

[17] Dolmaci OB Legué J Lindeman JHN Driessen AHG Klautz RJM Van Brakel TJ The extent of coronary artery disease in patients with stenotic bicuspid versus tricuspid aortic valves. *J Am Heart Assoc.* (2021) 10:e020080. 34075785 10.1161/JAHA.120.020080 PMC8477872

[18] Dolmaci OB Driessen AHG Klautz RJM Poelmann R Lindeman JHN Grewal N. Comparative evaluation of coronary disease burden: bicuspid valve disease is not atheroprotective. *Open Heart.* (2021) 8:e001772. 10.1136/openhrt-2021-001772 34497063 PMC8438949

[19] Grewal N Gittenberger-de Groot AC Lindeman JH Klautz A Driessen A Klautz RJM Normal and abnormal development of the aortic valve and ascending aortic wall: a comprehensive overview of the embryology and pathology of the bicuspid aortic valve. *Ann Cardiothorac Surg.* (2022) 11:380–8. 10.21037/acs-2021-bav-14 35958528 PMC9357963

[20] Grewal N Gittenberger-de Groot AC Thusen JV Wisse LJ Bartelings MM DeRuiter MC The development of the ascending aortic wall in tricuspid and bicuspid aortic valve: a process from maturation to degeneration. *J Clin Med.* (2020) 9:908. 10.3390/jcm9040908 32225051 PMC7230962

[21] Sinha S Hoofnagle MH Kingston PA McCanna ME Owens GK. Transforming growth factor-beta1 signaling contributes to development of smooth muscle cells from embryonic stem cells. *Am J Physiol Cell Physiol.* (2004) 287:C1560–8. 10.1152/ajpcell.00221.2004 15306544

[22] Halushka MK Angelini A Bartoloni G Basso C Batoroeva L Buja LM Consensus statement on surgical pathology of the aorta from the Society for Cardiovascular Pathology and the Association For European Cardiovascular Pathology: iI. Noninflammatory degenerative diseases–nomenclature and diagnostic criteria. *Cardiovasc Pathol.* (2016) 25:247–57. 10.1016/j.carpath.2016.03.002 27031798

[23] Toma I McCaffrey TA. Transforming growth factor-β and atherosclerosis: interwoven atherogenic and atheroprotective aspects. *Cell Tissue Res.* (2012) 347:155–75. 10.1007/s00441-011-1189-3 21626289 PMC4915479

